# Flavor Profiling by Consumers Segmented According to Product Involvement and Food Neophobia

**DOI:** 10.3390/foods10030598

**Published:** 2021-03-12

**Authors:** Yun-Mi Lee, Seo-Jin Chung, John Prescott, Kwang-Ok Kim

**Affiliations:** 1 Department of Food Science and Engineering, Ewha Womans University, Seoul 03760, Korea; insideyunmi@naver.com; 2 Department of Nutritional Science and Food Management, Ewha Womans University, Seoul 03760, Korea; sc79d@ewha.ac.kr; 3 Taste Matters Research & Consulting, Sydney 1230, Australia; Prescott@taste-matters.org

**Keywords:** consumer, sensory profiling performance, intensity, involvement, food neophobia

## Abstract

The relationship between food-related individual characteristics and performance in sensory evaluation was investigated. The study focused on differences in discriminative ability and perceptual sensitivity according to levels of product involvement or food neophobia during the intensity rating of sensory attributes in consumer profiling. Consumers (N = 247) rated the intensity of attributes for seven flavored black tea drinks and completed the Food Neophobia Scale and the Personal Involvement Inventory measuring product involvement with the flavored black tea drink. In the higher product involvement (IH) group and the lower food neophobia (NL) group, the number of sensory attributes representing the sample effect and of subsets discriminating the samples were greater, and more total variance of the samples was explained. The higher the product involvement or the lower the food neophobia, the greater the differentiation in characterizing samples with more attributes in the intensity ratings. Interestingly, the high food neophobia (NH) group showed less active performance compared to the NL group during the sensory evaluation overall, but the NH group was more concerned about unfamiliar attributes and samples. The results implied that the positive attitude resulting from high product involvement and low food neophobia may induce more active behavior and better performance during the sensory evaluation.

## 1. Introduction

Sensory descriptive tools using untrained panelists/consumers have recently received attention as legitimate alternatives to the application of conventional sensory analytical tests. These novel tools, including free choice profiling (FCP) [1], sorting [2], flash profiling (FP) [3] napping^®^ [4], check-all-that-apply [5], and rate-all-that-apply (RATA) [6], are both more rapid and easier to implement by untrained panelists than conventional analytical tests [7]. More importantly, sensory profiling by consumers may provide additional, important information that can elucidate consumers’ perceptions of products [8,9]. Therefore, sensory profiling methods using untrained panelists are considered extremely useful, even though there is some concern that consumers may not provide the detailed, robust, and reproducible results typical of methods that use trained panelists [10].

It has been suggested that errors that occur due to consumers’ unfamiliarity with methods or products can be prevented by providing detailed instructions [9] or that these shortcomings could be resolved statistically [11]. However, a different perspective is that the supposed weaknesses of consumer profiling are not problems to be solved, but rather represent pertinent issues to be understood [12,13,14,15,16,17,18]. Therefore, a more appropriate way of considering the variability in data from consumer sensory profiling is to interpret the results in the light of measured individual differences between consumers, including demographic, geographic, psychological, and behavioral variables related to the product of interest. Thus, it is important to accompany such forms of sensory profiling with consumer classification or segmentation [19,20], with subsequent analysis treating consumer characteristics as explanatory or predictive variables. Accordingly, the criteria for classification should be carefully considered so that the results can show more clearly how consumers perceive the product.

In the present study, two factors were applied to better understand the result of consumer profiling. One is product involvement, known to be an important characteristic affecting consumers’ attitudes and behaviors towards foods. Involvement is defined as the relevance that a product has for the consumer [21], and it is related to active attitudes aimed at understanding product information [22]. Another factor, food neophobia, traditionally refers to the degree of reluctance that a consumer has in trying novel foods [23]. These two factors can be considered as representative food-related personality traits [24]. Recent studies have shown that differences in food neophobia predict the consumption and acceptability of foods generally, including foods that may be quite familiar [25,26,27,28], but that food is rejected for reasons other than novelty, e.g., by uncommon flavor combinations.

We suggest, therefore, that consumers will show different degrees of attention or activeness in processing the sensory properties of food products, depending on how involved they are in the food product or the extent to which the product elicits food neophobia, and that this will be reflected in the performance of sensory evaluation. Some studies evoked negative emotions toward a product and better discrimination of the samples was observed [29,30]. Particularly, this study focused on whether consumers show differences in discriminative ability or perceptual sensitivity according to their product involvement or food neophobia level during the intensity rating of sensory attributes in consumer profiling of black tea as the model system. So far, there have been few studies on differences in flavor intensity ratings by consumers with different levels of food product involvement and food neophobia. In the case of sweetness, saltiness, and overall strength of flavor, people with higher levels of involvement (especially in part of the ‘preparation and eating involvement’ subscale) showed greater discrimination among samples [31]. Therefore, the present study was aimed at exploring the criteria for consumer classification, or the factors for interpretation of sensory profiling, hypothesizing that consumers who are highly involved with the product and food neophobics will show better discrimination in sensory profiling. Black tea was chosen as the model beverage in the study since it is a familiar beverage among Korean consumers, but applying different types of flavor to black tea can induce different degrees of novelty/unfamiliarity in black tea perception.

## 2. Materials and Methods

### 2.1. Samples

#### 2.1.1. Materials

Flavored black tea drinks, made by adding various flavored syrups (MONIN Asia KL Sdn Bhd, Selangor, Malaysia) to the Assam black tea base (Tea Korea Co., Ltd., Bucheon, Korea), at various concentrations, were used for intensity evaluation by consumers. The amount of black tea base, water, and flavored syrups used was based on the recommendations on the package of the products and on preliminary testing.

#### 2.1.2. Sample Design

The flavors of the syrups to make the flavored black tea samples in this study were peach, apple, melon, and cucumber. Peach is the most frequently used flavor in commercial black tea drinks, whereas cucumber has never been applied to black tea in Korea. Thus, peach and cucumber syrups were used as being familiar and unfamiliar flavors, respectively, in black tea drinks for Korean consumers. Apple and melon syrups were also used as other relatively familiar flavors.

In order to prevent large difference in the characteristics of the samples, all the drink samples contained peach syrup plus either one or two other syrups at lower concentrations (*w*/*w*, 2.7% of the sample) than the peach syrup (*w*/*w*, 5.4% of the sample), except one sample (UU) which had no other flavoring syrup but peach. The seven sample flavor combinations are as follows (Table 1): UU, no other syrup; AU, apple; MU, melon; CU, cucumber; AM, apple and melon; MC, melon and cucumber; CA, cucumber and apple. To make the total sugar content of seven samples similar, unflavored sugar cane syrup (MONIN Asia KL Sdn Bhd, Selangor, Malaysia) was used. °Brix range among the samples was from 7.27–7.40 (RHB-32ATC, Huake Instrument Co., Ltd., Shenzhen, China).

#### 2.1.3. Sample Preparation and Presentation

Aliquots of each sample (50 mL) were poured into opaque plastic cups (160 mL, Easepack Co., Namyangju, Korea) coded with three-digit random numbers. The samples were served simultaneously at room temperature (20 ± 2 °C). The presentation order of the seven samples followed a Williams Latin Square Design [32]. Each participant was provided with filtered water (Ceramic Filter System, Supercape, Dalton, Fairey Industrial Ceramics Ltd., London, UK) and was instructed to drink it for mouth rinsing between samples.

### 2.2. Taste and Flavor Descriptors

Sensory descriptors were generated by 31 untrained (with no experience in descriptive analysis; aged 19–70; 12 males and 19 females) and nine trained panelists (with experience in descriptive analysis; aged 24–42; nine females) using samples with three flavor syrups in black tea at various concentrations. Untrained panelists used seven words on average (range from 2–23 words) to describe the samples, while trained panelists elicited 18 attributes on average (range from 11–25). A total of 87 and 91 attributes related to taste/flavor modality were generated for the samples by untrained and trained panelists, respectively. The trained panelists mostly derived descriptive terms that represent distinct flavors. The untrained panelists, however, used more comprehensive descriptors such as category and name of the product or food, and they expressed overall impressions of taste/flavor such as fruity, flat, and watery.

Based on the list obtained from the untrained panelists, the attributes were selected considering the frequency of usage employed by two or more panelists. Focused on taste/flavor attributes, the recognizable attributes without need for interpretation (objective descriptions) were finally selected (flavors: apple, peach, melon, cucumber, and plum; tastes: sweet, sour, and bitter). In order to select more representative attributes of black tea samples, the attributes generated by trained panelists were considered. Given that the samples were made from black tea base, “astringent” and “black tea flavor” were also included, resulting in a final list of 10 attributes (sweet, sour, bitter, apple, peach, melon, cucumber, plum, black tea, and astringent).

### 2.3. Subjects

A total of 245 subjects who had previously consumed flavored black tea drinks were recruited through on/off-line advertisement in Seoul, Korea. They reported no experience in descriptive analysis and no food related allergies. Demographics of subjects (gender, age, and education) are shown in Table 2. The subjects consisted of 40.4% male and 59.6% female. The mean age was 31 years (SD = 11.66, range 13–73), and the ratio of low, medium, and highly educated subjects were 22.0, 31.8, and 46.1%, respectively: low, ≤high school; medium, undergraduate; high, graduate.

### 2.4. Procedure

Samples were evaluated monadically. Subjects rated the intensities of the ten pre-established sensory attributes on 15-point category scales, from “weak (1) to “strong (15)” after tasting each sample. Subjects were instructed to taste and swallow the samples freely. After completing the evaluation of one sample, they were asked to drink filtered water (20 ± 2 °C) to rinse their palate and have a 1 min break before tasting the next sample.

To classify subjects who participated in this study according to their individual traits, subjects were required to complete the Personal Involvement Inventory (PII) [33,34] regarding flavored black tea drinks and the Food Neophobia Scale (FNS) [23]. For the analysis, they were classified into low, moderate, and high product involvement (PI) groups and low, moderate, and high food neophobia (FN) groups according to the level of product involvement and food neophobia, respectively. The PII is composed of 15 questions such as ‘important’, ‘of concern to me’, ‘relevant’, ‘mean a lot to me’, ‘interesting’, etc., to measure the perceived relevance of the product to each person. For the subject classification based on the sum of PII scores, subjects of 25% from top and bottom percentile ranks respectively were taken as high and low involvement group (called IH and IL) following the method as in a previous study [34]. The subjects in the middle of the score distribution were designated as the moderate involvement group (IM). High and low food neophobia groups (called NH and NL) were allocated above the mean plus standard deviation of summed FNS scores and below the mean minus standard deviation of summed FNS [23]. The subjects with intermediate scores were also designated as the moderate food neophobia group (NM).

Tests were conducted on campus, in churches, or in the community rooms in Seoul, Korea. The study was approved by the institutional Review Board (IRB) at Ewha Women’s University, Seoul, Korea (#138).

### 2.5. Data Analysis

Three-way analysis of variance (ANOVA) with Product involvement (low; moderate; high levels), Food neophobia (low; moderate; high levels), and Samples as factors was carried out on the intensity rating data in order to check the overall presence of their main effect and interactions. In addition, post-hoc comparisons using the Tukey’s HSD test (α = 0.05) were performed on the intensity rating data to compare the differences among the groups in the sensory evaluation. In order to summarize the interrelationship among descriptors and samples, principal component analysis (PCA) was conducted on the averaged data. Additionally, multiple factor analysis (MFA) was performed to examine the similarity of sample positioning between comparative groups. Statistical analyses were performed using PASW Statistics 18.0 (SPSS Inc., Chicago, IL, USA) and XLSTAT for windows 14.0 (Addinsoft, rue Damrémont, France).

## 3. Results

### 3.1. Subject Groups According to Product Involvement Level and Food Neophobia Level

**Figure 1:**
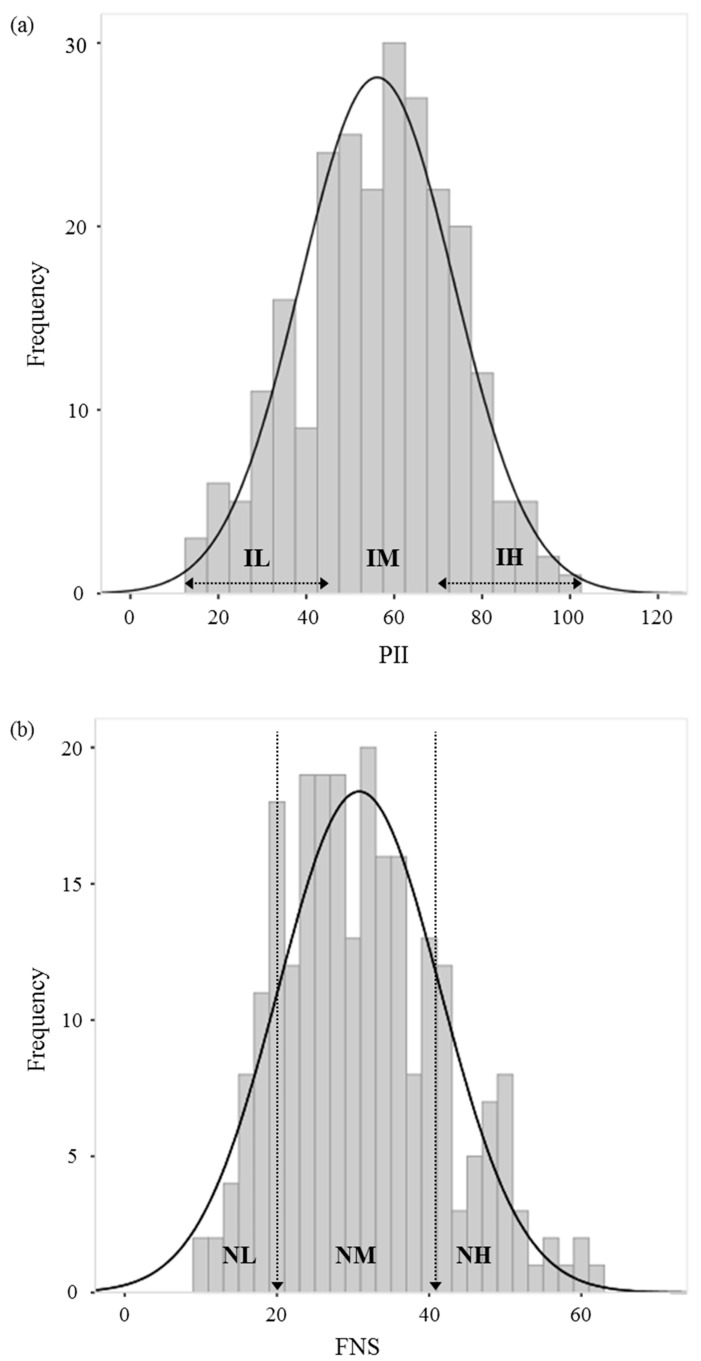
The distribution of the Personal Involvement Inventory (PII) (**a**) and the Food Neophobia Scale (FNS) (**b**) scores.

Participants’ (N = 245) PII scores ranged from 15 to 99 for the flavored black tea drink (Figure 1a). The mean scores of low involvement (IL), moderate involvement (IM), and high involvement (IH) groups were 32.98 (N = 61; range: 15 to 44), 56.45 (N = 117; 45 to 67), and 76.57 (N = 67; 68 to 99), respectively. The mean FNS score of all subjects (N = 245, Figure 1b) was 30.81 (SD = 10.63; ranged from 10 to 61). The criteria for low food neophobia (NL) and high food neophobia (NH) group were scores on the FNS below 20.18 and above 41.44, respectively. The mean scores of NL, NM (moderate food neophobia), and NH groups were 17.07 (N = 45; range: 10 to 20), 30.40 (N = 162; 21 to 41), and 48.84 (N = 38; 42 to 61), respectively.

### 3.2. Effects of Samples

There were significant (*p* < 0.05) sample effects on the intensity ratings of sour (*F*_6,1648_ = 5.77), apple (*F*_6,1645_ = 12.21), peach (*F*_6,1645_ = 6.47), melon (*F*_6,1648_ = 39.45), cucumber (*F*_6,1649_ = 31.14), plum (*F*_6,1645_ = 6.22), and black tea (*F*_6,1646_ = 6.27) attributes, but not for the sweet, bitter, and astringent attributes. The mean intensity of sour was relatively high in AM, and low in CU and UU. Apple flavor intensity was highest in AU, and low in the samples in the order of MU > CA > CU > MC. Peach flavor intensity was high in UU and AU, and low in MC. The intensity of cucumber flavor was high in CU, MC, and CA, and melon flavor intensity was also high in MC and CA, while, the intensities of both attributes was low in UU and AU. Especially in cucumber flavor, the samples were separated into three groups depending on the addition of cucumber and melon syrup. Plum flavor intensity was relatively high in AU and AM, and low in MU, MC, and CU. The intensity of black tea flavor was highest in UU and relatively low in MC. Consequently, UU was characterized by peach and black tea flavors, and AU was highlighted by apple, peach, and plum flavors while CU was mainly characterized by cucumber flavor. MC and CA were explained by melon and cucumber flavor while AM was recognized by sour and plum attributes.

### 3.3. Effects of Product Involvement (PII)

**Figure 2:**
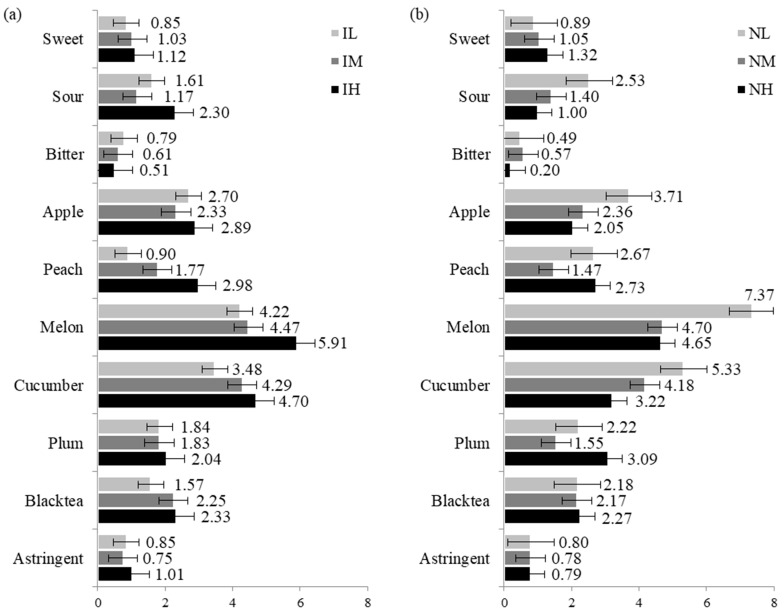
The range magnitude of the group mean value for each level of the (**a**) PII and (**b**) FNS measures for the intensities of the attributes: the maximum value minus the minimum value within each attribute and groups. Bars represent standard error.

**Figure 3:**
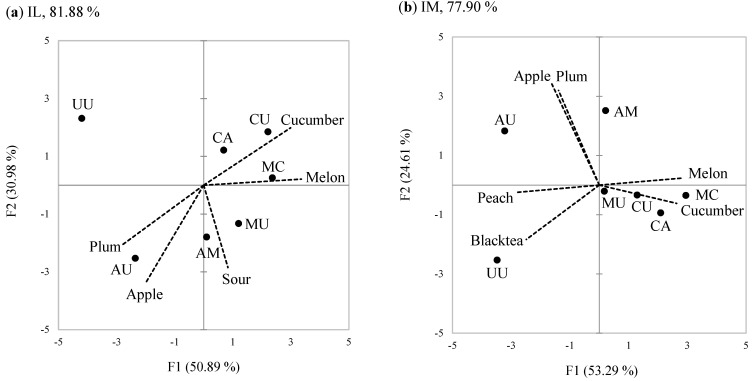

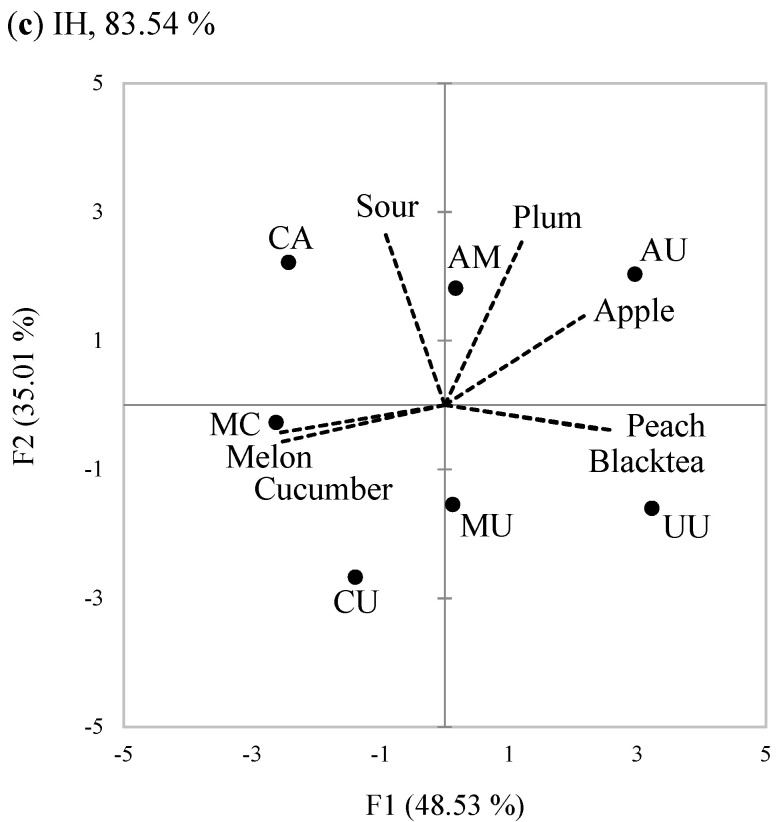
Principal Component Analysis (PCA) biplot of the samples (filled circles) and the sensory attributes (dotted line) for the first two PC dimensions: (**a**) IL; (**b**) IM; (**c**) IH. See Table 1 for sample codes. Only the attributes showing significant differences among the samples (*p* < 0.05) are presented.

There were significant (*p* < 0.05) main effects of product involvement on the intensity rating of all attributes. Post-hoc comparisons of PI groups were differentiated into post-hoc subgroups for all the attributes, with the exception of astringency. As shown in Figure 2a, the IH group further distinguished the samples using a wider range of the scale than the IL group on all attributes except bitterness. The rating-range magnitude of the IM group was intermediate between the IL and IH groups, with the exception of sour and apple attributes. This rating-range magnitude may imply the degree of effort or attention when trying to differentiate the sensory characteristics of the samples. The PCA on the IL group (Figure 3a) showed that the first two PCs explained 50.89% and 30.98% of the total variance (81.88%), respectively. Considering only the attributes with significant sample effects, the peach flavored black tea drink (the base) containing melon or cucumber syrup (MC, CU, MU, CA, and AM) were loaded on the positive PC1, and were rated relatively high in melon and cucumber attributes (Figure 3a, Table 3). On the negative side of PC1, only those including peach and/or apple syrups (UU and AU) were loaded, and were characterized as plum attribute. On PC2, apple and sour attributes characterized the samples having apple and/or melon syrup (AU, AM, and MU), differentiating those having cucumber syrup (CU, CA, and MC) or no added syrup (UU) from the base.

In the IH group (Figure 3c), a total of 83.54% of the variance was explained by PC1 (48.53%) and PC2 (35.01%). The two samples having only peach and/or apple syrup (UU and AU) were highly loaded on the positive PC1 with peach, black tea, and apple attributes. In contrast, the samples containing cucumber syrup in the base such as MC, CA, and CU were positioned on the negative PC1 with melon and cucumber attributes. In addition, the rest of the samples (MU and AM), having melon syrup but no cucumber syrup, were positioned in the middle of PC1 axis, indicating that IH group differentiated MU and AM from the other samples. This means that the IH group differentiated the samples better than did the IL group on the axis of PC1. On PC2, the IH group placed the samples containing apple syrup (CA, AU, and AM) on the positive PC2, characterized them with sour and plum attributes, and positioned the others (CU, MU, UU, and MC) not containing apple syrup on the negative PC2. Accordingly, the IH group explained a greater total variance of samples than did the IL group. The IM group (Figure 3b) explained the lowest total variance (77.90%) of the first two PCs than the other groups. In the IM group, the samples (MU, CU, CA, and MC), positioning only in the second quadrant, were less distinguished and less explained than in the IH group. On the positive side of PC2, they did not distinguish differences between apple and plum attributes while the IH group did.

**Figure 4:**
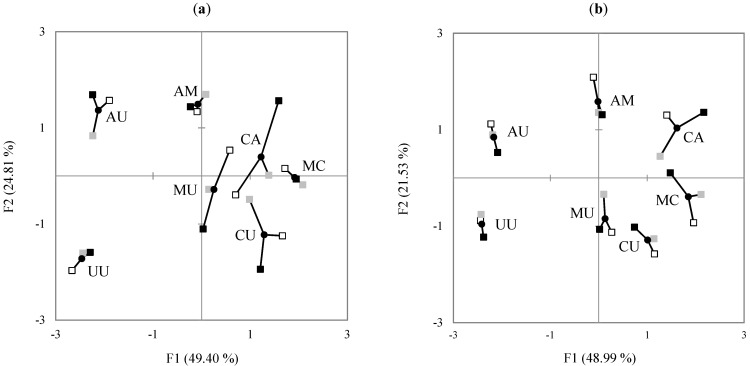
Superimposed representation of the samples (●) from multiple factor analysis (MFA): (**a**) IL, 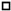; IM, 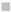; IH, 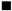, (**b**) NL, 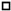; NM, 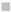; NH, 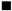. See Table 1 for sample codes.

The MFA on the mean intensity value of the attributes also showed that the IH group better distinguished the samples in sensory intensity ratings than the IL group (Figure 4a). In particular, the IH group perceived the difference between MU and CA as much greater than did the IL group. It seems that IH group distinguished between the samples including melon syrup and the samples including cucumber syrup while the IL group was confused by these samples. Though cucumber and melon flavors might be expected to be confused, since other fruit flavors and sweetness were added, the IH group distinguished whether the samples contained cucumber and melon syrup, or not, and characterized more attributes. Furthermore, the IH group seemed to recognize the difference in the presence or absence of apple syrup. Unlike the IL group, the IH group distinguished the samples containing apple syrup from the samples without apple syrup and characterized them as having sour and plum attributes.

### 3.4. Effects of Food Neophobia (FNS)

**Figure 5:**
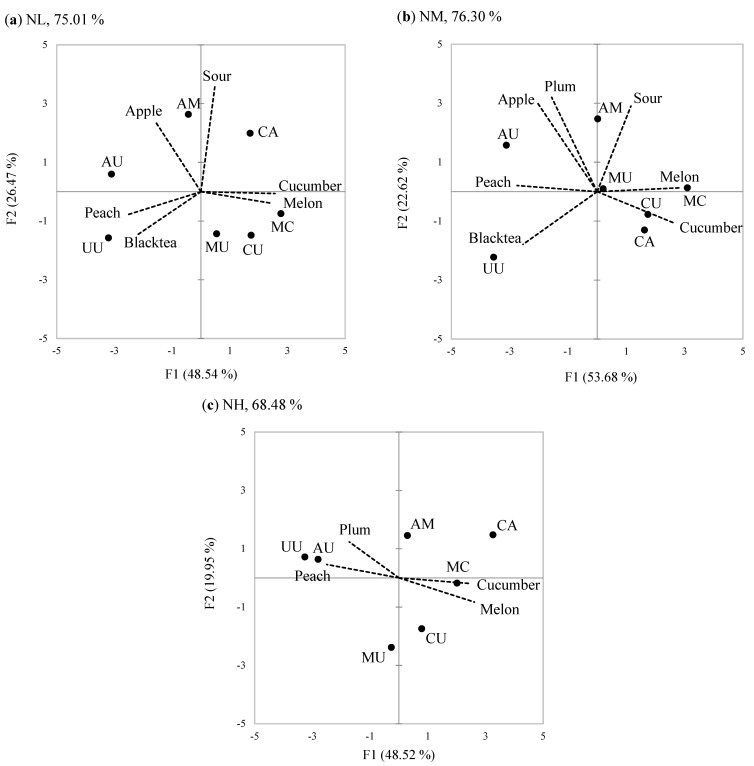
PCA biplot of the samples (filled circles) and the sensory attributes (dotted line) for the first two PC dimensions: (**a**) lower food neophobia (NL); (**b**) moderate food neophobia (NM); (**c**) high food neophobia (NH). See Table 1 for sample codes. Only the attributes showing significant differences among the samples (*p* < 0.05) are presented.

There were significant (*p* < 0.05) main effects of Food neophobia on the intensity ratings of all attributes. In the post-hoc comparisons of mean intensity scores, FN groups were differentiated into different homogeneous subsets for all attributes. The PCA result showed that PC1 and PC2 explained 48.54% and 26.47%, respectively, of total variance (75.01%) in NL group (Figure 5a). Considering only the attributes with significant sample effects, those having cucumber syrup such as MC, CU, and CA were highly loaded on the positive PC1 and they shared melon and cucumber attributes. On the negative PC1, there were high loadings for samples containing only peach and/or apple syrup (UU and AU), which were characterized with peach and black tea. On PC2, AM, CA, and AU, which had apple syrup, were loaded positively and separated from other samples. The NM group (Figure 5b) explained a similar percentage of the total variance (76.30%) to the NL group. Both NL and NM groups showed well which attributes explained the samples and how much the attributes represent the sample on the PCA plot. However, the NM group did not identify apple flavor in CA, and the NL group did not explain the samples by plum attribute.

In the NH group, the first two PCs accounted for 48.52% and 19.95% of total variance, respectively (Figure 5c), explaining lower total variance than in the NL group. Cucumber syrup samples CA, MC, and CU were loaded positively on PC1 and characterized by melon and cucumber attributes. Peach syrup and/or apple syrup samples UU and AU were loaded negatively on PC1 and were highly correlated with peach and plum attributes. Compared to the NL group, the NH group explained less total variance in the PCA, and did not differentiate the samples on several attributes (sour, apple, and black tea), thus indicating a lower overall performance of the NH group. Although both groups distinguished the samples containing cucumber syrup, the distance among the samples containing cucumber and/or melon syrup in the NH group were greater than that in the NL group, suggesting that the NH group may have focused more on the difference between these two attributes than the NL group. These performances are also confirmed from the superimposed representation of partial groups. As shown in Figure 4b, overall, the NL group tended to differentiate the samples more than the NH group. The exception to this was the sample CA, which was greatly differentiated by the NH group who rated this sample as having the strongest cucumber and melon characteristics. However, the NL group used a wider range of scale values than did the NH group for the intensities of sour, apple, melon, and cucumber attributes (Figure 2b), which were the main contributors to the PC loadings in both groups (Figure 5). For the black tea attribute, the three FN groups used a similar range magnitude of the scale. For peach and plum attributes, however, the NM group used a narrower range of the scale than the other groups.

### 3.5. Product Involvement and Food Neophobia Interactions

**Figure 6:**
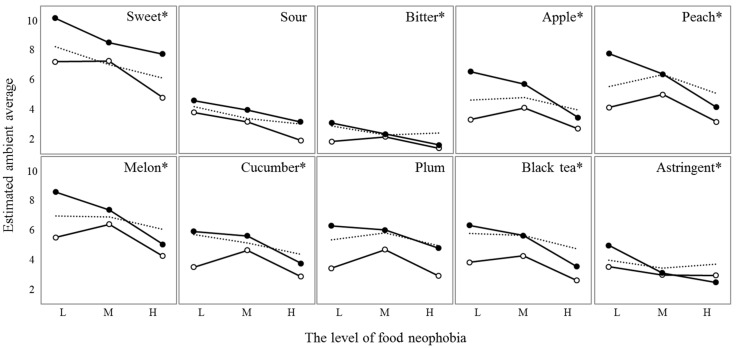
Plots of the interaction effects between Product involvement (low, ○; moderate, dotted line; high, ●) and Food neophobia (*x*-axis: low, L; moderate, M; high, H) for each sensory attribute. The *y*-axis values represent the estimated ambient averages of intensity ratings for each case. An asterisk indicates a significant interaction effect (*p* < 0.05) for each sensory attribute.

There were significant (*p* < 0.05) Product involvement and Food neophobia interactions for all attributes except sour and plum (Figure 6). The significant interactions resulted from large differences in intensity ratings depending on the level of involvement, especially in the case of low food neophobia. The participants with high product involvement and low food neophobia gave higher intensity ratings to all the sensory attributes than did other groups. There was a significant interaction between Food neophobia and Samples (*F*_12,1648_ = 1.98, *p* = 0.02) on the intensity rating of melon attribute. However, there were no significant interactions between the other combinations of ANOVA factors (Product involvement × Samples, Food neophobia × Samples, Product involvement × Food neophobia × Samples) on the intensity rating of all attributes.

Looking back at the sample effect by group, the effects of samples were shown to be relatively greater in the IH and NL group with larger *F*-values (data not shown) than in the IL and NH group, respectively. According to the post-hoc comparisons of mean intensity scores within each group, the subgroups for the IH and NL groups were more similar to those for the total subjects than the IL and NH groups. In three PI groups, the number of post-hoc subgroups was different between the IL, IM, and IH groups in the intensity ratings of apple, peach, cucumber and black tea attributes (Table 3). Especially for peach and black tea attributes, i.e., the major attributes of the black tea drinks, the IH group distinguished the samples with more subgroups than the IL and IM groups. For apple attribute, the IM group distinguished the samples with more subgroups than the other groups, but the subgroups for the IH group were more similar to those for the total subjects than the other groups. In the case of cucumber attribute, though the IL group separated the samples into many more subgroups than the other groups, the IH group correctly distinguished between the samples containing cucumber syrup (CU, MC, and CA) and those that did not (UU, AU, MU, and AM). In the FN groups, the number of post-hoc subgroup was different in the intensity ratings of apple and plum attributes (Table 4). In the intensity evaluation for apple attribute, the NL group rated the samples containing apple syrup (AU, AM, and CA) a high score and distinguished them from other samples. The NM and NH groups also knew the sample with the most obvious apple flavor (AU), but the NM group was confused about its intensity, and the NH group did not distinguish the samples according to the intensity of apple flavor. Like the IH group among three PI groups, the NL group exactly distinguished the presence of cucumber syrup in the samples (CU, MC, and CA).

## 4. Discussion

This study aimed to test factors that previous studies have suggested [29,30,31] might affect consumer performance in the intensity rating of sensory attributes. In particular, the study focused on the individual consumer characteristics that influence the attitudes toward food, namely levels of product involvement and food neophobia. This study is noteworthy given the lack of studies on the relationship between individual characteristics and behaviors during sensory profiling, especially in regard to food product involvement and neophobia. It therefore provides data that can support improved performance in consumer sensory profiling studies. Although only beverages were evaluated, this study used products with which the participants—females, young adults, and the highly educated—were highly involved, namely flavored black tea drinks. The results indicated that the subjects with the highest product involvement or low food neophobia characterized and differentiated the samples to a greater degree. It should be noted, however, that the results of this study cannot guarantee that the IH and NL groups have better sensory sensitivity than the IL and NH groups, respectively. Instead, it is likely that the IH and NL groups make a greater effort or pay greater attention when trying to differentiate the sensory characteristics of the products. That is, the IH and NL groups may perform better sensory discrimination motivated by their emotional involvement. Hence, in the study of Stocks et al. [35], authenticity stories, that engaged emotional involvement, led to better discrimination among barely discriminable stimuli.

Previous studies have shown that individual consumer characteristics/traits influenced attitudes [36,37,38], and that these attitudes were mostly consistent with behavior [39]. Given that individual difference factors such as product involvement and food neophobia can influence attitudes toward the food product, it is quite clear that positive attitudes resulting from high product involvement and low food neophobia can lead to subjects’ greater discriminative ability during the sensory evaluation. For instance, Ares et al. [40] showed that the individuals’ product involvement affects the interest in the evaluated samples and reaction towards the sensory variables of samples. Given that food neophilia groups show higher willingness to try unfamiliar foods and higher consumption of the samples than food neophobia groups [41], the NL group in this study seemed to have a more positive attitude towards their performance in the sensory evaluation task.

Generally, the NH group had been expected to show greater discriminative ability in the sensory evaluation and to differentiate the samples to a greater degree than the NL group, since those high in food neophobia are known to avoid unfamiliar foods [23] and therefore should be more sensitive to unfamiliar products. However, the NL group showed better discriminative ability during the sensory rating than the NH group. Although both groups had a tentative motive to show greater discriminative ability during the sensory evaluation, it seemed that the performance (active behavior) induced by the acceptance (positive motivation) of the NL group was greater than the performance induced by the rejection (negative motivation) of the NH group in the sensory evaluation of the flavored black tea drinks. It is also supported by previous studies showing that those low in FN had a more positive attitude toward, and experiences with, a variety of foods [23,42].

In this study, groups varying in product involvement or food neophobia showed differences in scale use during the intensity rating. In order to accurately identify differences among samples in sensory science, there have been efforts to validate sensory data analysis in relation to the differences in panels’ scale use [43,44,45]. For instance, in studies of the mixed model [3,9,46,47,48,49], individual differences within the panel, and interactions between panel and product were considered as random effects in statistical analysis. Another statistical approach to handle these individual differences is principal component analysis (PCA). In PCA, differences in scale use among panels are dealt with by averaging scores over panels [50]. In descriptive analysis with trained panelists, training procedures reduce the effect of individual differences of the panelists.

In contrast, individual differences within panels of consumers are important in that they reflect the individual consumer’s natural perception of the product. Individual differences that arise from sex or age are routinely considered in consumer studies. What the present study shows is that other individual consumer traits—specifically, product involvement and food neophobia—can be equally important in determining sensory evaluation outcomes. Previous research supports this conclusion. Lim [51] indicated that psychological traits can affect sensory evaluation and decision making. For instance, the level of motivation according to individual traits can psychologically influence the scores [52]. To some extent, therefore, differences within consumer panels in sensory evaluation results might be explained on the basis of such individual traits.

Despite this study’s contribution in identifying the effect of product involvement and food neophobia on the intensity rating of sensory attributes, it has some limitations. The participants were not verified as representative of the entire population even though the subjects who participated in this study were relatively varied in terms of gender, age, and education. The comparisons of the PII scores by subject factors such as gender, age, and education in previous studies showed inconsistent results depending on the product of interest; for instance, in gender, a highly involved group for chocolate desserts was composed of a high proportion of females [40], and highly involved people in sausages and chocolate bars were mainly males and females, respectively [53]. Although it is difficult to compare the mean FNS scores by country since the number, age, and gender of participants in each study are diverse, Koreans [54,55] generally tend to be slightly higher in mean food neophobia than the US population [56] and lower than other populations [23,57]. The gender ratio of food neophobia in this study showed a similar result to several previous studies [57,58,59]. However, the relationship between food neophobia and gender has not been clarified yet since there are also studies reporting no gender effect on food neophobia [23,56,60,61,62,63]. The higher the education level, the lower the FNS score (*p* < 0.05) in the present study, which is the same as in the studies that reported the negative correlation between FNS score and education level [57,64,65].

To some extent, the results of this study are limited by the fact that the acceptability of the samples was not measured. Given that there are previous studies supporting the correlation between involvement and interest [26] and between food neophobia and acceptability [25,55], the overall acceptability of the samples might provide additional evidence or information about the relationship between individual characteristics and performance in sensory evaluation. Nevertheless, since it is not desirable for the same subjects to undertake analytic profiling and judge holistic acceptance within the same test [66], overall acceptability was not evaluated in this study. To some degree, familiarity can be considered a proxy for acceptability. Here, familiarity was measured, with the peach flavored black tea drink being the most familiar (33.2% of the total subjects answered ‘extremely familiar’), followed by apple (18.6%, ‘familiar’), melon (22.7%, ‘slightly unfamiliar’), and cucumber (40.5%, ‘extremely unfamiliar’). Since there was a gradient of sample familiarity from ‘extremely familiar’ to ‘extremely unfamiliar’, it can be assumed that the samples differed in acceptability to some degree [41].

In conclusion, this study suggests that there is an important relationship between food-related consumer characteristics and performance in sensory evaluation. The differences in discriminative ability and perceptual sensitivity were shown according to the levels of product involvement and food neophobia during the intensity rating of sensory attributes. Given that high product involvement or low food neophobia induced sensitive discrimination as shown in this study, the level of product involvement and food neophobia can be used not just to understand the results of consumer sensory profiling, but also to improve the performance of consumers during sensory profiling. The practical implication of the present study is therefore related to making sensory studies with consumers more effective. For example, screening consumers based on the level of product involvement and food neophobia prior to sensory profiling may possibly reduce the amount of training required for consistent and accurate sensory profiling by improving the evaluation performance. It will be interesting to investigate the effectiveness of using panelists with high PI or low neophobia in future research.

## Figures and Tables

**Table 1:** Information on the seven flavored black teas used for intensity rating of sensory attributes.

Sample Code	Features				
	Composition			°Brix (%)	
	High	Low	Low	Mean	(±SD)
UU	Peach	Unflavored	Unflavored	7.27	(0.12)
AU	Peach	Apple	Unflavored	7.40	(0.00)
MU	Peach	Melon	Unflavored	7.40	(0.00)
CU	Peach	Cucumber	Unflavored	7.40	(0.00)
AM	Peach	Apple	Melon	7.40	(0.00)
MC	Peach	Melon	Cucumber	7.40	(0.00)
CA	Peach	Cucumber	Apple	7.33	(0.12)

All the samples contained a higher concentration of peach syrup (*w*/*w*, 5.4%) than other syrup. The other syrups were used at lower concentration (*w*/*w*, 2.7%).

**Table 2:** Product involvement and food neophobia scores by gender, age, and education.

Variable	Categories	Involvement ^1^		Neophobia ^2^		N
		Mean	Range	Mean	Range	
Gender	Male	52.00	15–88	29.12	11–61	99
	Female	58.90	15–99	31.95	10–60	146
Age groups	10–19 yrs	51.41	15–99	34.25	15–60	32
	20–29 yrs	59.21	18–96	29.22	10–61	120
	30–39 yrs	56.98	21–93	29.06	12–56	50
	40–49 yrs	50.43	18–74	34.19	20–51	21
	50–59 yrs	50.86	27–87	34.93	24–52	14
	60 yrs+	47.13	15–88	35.63	13–58	8
Education ^3^	Low	47.83	15–99	36.15	13–61	54
	Medium	61.53	18–96	32.38	19–60	78
	High	56.33	21–90	27.17	10–56	113

^1^ Personal involvement inventory [33,34]; ^2^ Food neophobia scale [23]; ^3^ Low, ≤ high school; Medium, undergraduate; High, graduate.

**Table 3:** Mean intensity scores for sensory attributes ^1^ of the samples in low involvement (IL), moderate involvement (IM) and high involvement (IH) groups.

Attributes	Group	Samples ^2^						
		No Other Syrup (UU)	Apple (AU)	Melon (MU)	Cucumber (CU)	Apple and Melon (AM)	Melon and Cucumber (MC)	Cucumber and Apple (CA)
Sweet	IL	7.48	6.92	6.66	6.62	6.72	6.8	6.95
	IM	7.58	7.64	7.19	7.41	6.95	6.61	6.62
	IH	9.16	8.73	9.1	9.18	8.48	8.06	8.34
Sour	IL	2.18 ^a^	3.20 ^ab^	2.83 ^ab^	2.26 ^a^	3.79 ^b^	3.25 ^ab^	3.43 ^ab^
	IM	2.87 ^a^	3.30 ^ab^	3.23 ^ab^	3.19 ^ab^	4.04 ^b^	3.38 ^ab^	3.79 ^ab^
	IH	2.88 ^a^	3.98 ^ab^	3.63 ^ab^	3.15 ^a^	4.64 ^ab^	3.55 ^ab^	5.18 ^c^
Bitter	IL	2.52	1.87	1.95	2.11	2	1.74	2.13
	IM	2.76	2.62	2.41	2.39	2.3	2.15	2.72
	IH	2.25	2.7	2.19	2.25	2.46	2.39	2.7
Apple	IL	3.67 ^a^	5.55 ^b^	3.87 ^a^	2.85 ^a^	4.08 ^ab^	3.13 ^a^	3.54 ^a^
	IM	4.31 ^ab^	6.36 ^c^	4.34 ^ab^	4.39 ^ab^	5.61 ^bc^	4.03 ^a^	4.13 ^a^
	IH	5.67 ^ab^	7.42 ^b^	5.27 ^a^	4.70 ^a^	6.27 ^ab^	4.53 ^a^	4.55 ^a^
Peach	IL	4.89	4.89	4.9	4.13	4.72	4.3	4
	IM	7.06 ^b^	6.62 ^ab^	6.21 ^ab^	5.70 ^ab^	6.09 ^ab^	5.35 ^a^	5.29 ^a^
	IH	7.88 ^c^	7.09 ^bc^	6.58 ^abc^	5.58 ^ab^	6.03 ^abc^	4.90 ^a^	5.40 ^ab^
Melon	IL	3.25 ^a^	3.52 ^a^	7.33 ^b^	7.08 ^b^	6.18 ^b^	7.47 ^b^	6.64 ^b^
	IM	4.01 ^a^	4.16 ^a^	7.91 ^b^	7.85 ^b^	7.29 ^b^	8.48 ^b^	8.13 ^b^
	IH	3.49 ^a^	3.54 ^a^	8.76 ^b^	8.72 ^b^	7.01 ^b^	9.40 ^b^	9.28 ^b^
Cucumber	IL	2.82 ^ab^	2.67 ^a^	3.79 ^abc^	6.15 ^d^	3.72 ^abc^	5.49 ^cd^	4.77 ^bcd^
	IM	3.03 ^a^	3.16 ^a^	4.68 ^b^	7.32 ^c^	4.52 ^ab^	6.59 ^c^	6.92 ^c^
	IH	3.19 ^a^	3.00 ^a^	4.52 ^a^	7.70 ^d^	4.90 ^c^	7.19 ^ab^	6.90 ^c^
Plum	IL	4.69 ^ab^	5.30 ^b^	3.59 ^ab^	3.46 ^a^	4.75 ^ab^	3.75 ^ab^	3.88 ^ab^
	IM	5.44 ^ab^	6.54 ^b^	5.50 ^ab^	5.04 ^a^	6.72 ^b^	4.89 ^a^	5.62 ^ab^
	IH	5.45 ^ab^	7.07 ^b^	5.57 ^ab^	5.03 ^a^	6.45 ^ab^	5.58 ^ab^	5.77 ^ab^
Black tea	IL	4.97	4.1	3.62	3.8	3.77	3.39	4.12
	IM	7.14 ^b^	5.91 ^ab^	5.46 ^a^	5.03 ^a^	5.32 ^a^	4.89 ^a^	5.36 ^a^
	IH	6.94 ^b^	5.85 ^ab^	5.18 ^ab^	4.97 ^a^	5.39 ^ab^	4.61 ^a^	4.81 ^a^
Astringent	IL	3.46	2.93	2.61	3.07	2.85	3.21	3.28
	IM	3.85	3.21	3.44	3.34	3.48	3.56	3.96
	IH	3.42	3.25	3.18	2.91	3.69	3.25	3.93

^1^ Group means: mean values within a row not sharing a superscript letter are significantly different (*p* < 0.05, Tukey’s HSD test). Attributes with non-significant differences among samples were not marked with superscript letters. ^2^ See Table 1 for sample codes.

**Table 4:** Mean intensity scores for sensory attributes ^1^ of the samples in NL, NM and NH groups.

Attributes	Group	Samples ^2^						
		UU	AU	MU	CU	AM	MC	CA
Sweet	NL	9.09	8.96	8.64	8.36	8.6	8.53	8.2
	NM	7.91	7.72	7.55	7.71	7.33	6.86	7.11
	NH	7	6.5	6.45	6.84	5.68	6.13	6.21
Sour	NL	3.2	4.22	3.48	3.16	5.3	4.02	5.69
	NM	2.65 ^a^	3.37 ^abc^	3.41 ^abc^	2.97 ^ab^	4.05 ^c^	3.44 ^abc^	3.83 ^bc^
	NH	2.32	2.92	2.24	2.61	3.21	2.45	3.24
Bitter	NL	2.93	2.71	2.64	2.93	2.96	2.47	2.71
	NM	2.58	2.51	2.24	2.25	2.15	2.05	2.62
	NH	2.05	1.92	1.74	1.68	1.95	1.95	2.18
Apple	NL	4.27 ^a^	7.49 ^c^	4.20 ^a^	4.07 ^a^	6.49 ^bc^	3.78 ^a^	4.84 ^ab^
	NM	4.81 ^ab^	6.56 ^c^	4.83 ^ab^	4.20 ^a^	5.50 ^bc^	4.27 ^a^	4.20 ^a^
	NH	3.63	4.79	3.3	3.66	3.74	2.74	2.76
Peach	NL	7.20 ^b^	6.80 ^ab^	6.42 ^ab^	5.42 ^ab^	5.76 ^ab^	4.53 ^a^	5.31 ^ab^
	NM	6.81 ^b^	6.59 ^ab^	6.25 ^ab^	5.58 ^ab^	6.00 ^ab^	5.48 ^a^	5.34 ^a^
	NH	5.87 ^b^	4.58 ^ab^	4.34 ^ab^	3.82 ^ab^	4.57 ^ab^	3.29 ^a^	3.14 ^a^
Melon	NL	3.51 ^a^	3.07 ^a^	10.44 ^b^	8.47 ^b^	7.16 ^b^	9.38 ^b^	8.09 ^b^
	NM	3.97 ^a^	4.19 ^a^	7.72 ^bc^	8.33 ^bc^	7.27 ^b^	8.67 ^c^	8.27 ^bc^
	NH	2.59 ^a^	3.24 ^a^	6.26 ^b^	5.37 ^ab^	5.29 ^ab^	6.66 ^b^	7.24 ^b^
Cucumber	NL	2.67 ^a^	2.78 ^a^	4.22 ^a^	8.00 ^c^	4.53 ^ab^	7.84 ^c^	6.98 ^bc^
	NM	3.35 ^ab^	3.15 ^a^	4.83 ^c^	7.33 ^d^	4.57 ^bc^	6.47 ^d^	6.48 ^d^
	NH	2.05 ^a^	2.58 ^ab^	2.87 ^abc^	5.26 ^c^	3.68 ^abc^	4.92 ^bc^	5.27 ^c^
Plum	NL	5.11	6.33	4.98	4.11	6.04	4.4	5.62
	NM	5.52 ^abc^	6.35 ^bc^	5.28 ^ab^	5.08 ^a^	6.59 ^c^	5.04 ^a^	5.48 ^abc^
	NH	4.32 ^ab^	6.51 ^b^	4.13 ^ab^	3.42 ^a^	4.42 ^ab^	4.29 ^ab^	3.68 ^a^
Black tea	NL	7.18	5.96	5.07	5.34	5	5.04	5.09
	NM	6.68 ^b^	5.58 ^ab^	5.22 ^a^	4.85 ^a^	5.19 ^a^	4.51 ^a^	5.31 ^a^
	NH	5.16	4.22	3.53	3.39	3.87	3.42	2.89
Astringent	NL	4.29	4.29	4.29	4.29	4.29	4.29	4.29
	NM	3.52	3.04	3.12	2.94	3.1	3.21	3.72
	NH	3.32	2.76	2.53	3.05	3.11	3.21	3.27

^1^ Group means: mean values within a row not sharing a superscript letter are significantly different (*p* < 0.05, Tukey’s HSD test). Attributes with non-significant differences among samples were not marked with superscript letters.^2^ See Table 1 for sample codes.

## Data Availability

Data available on request due to restrictions e.g., privacy or ethical.

## References

[1] Williams A.A., Langron S.P. (1984). The use of free-choice profiling for the evaluation of commercial ports. J. Sci. Food Agric..

[2] Lawless H.T., Sheng N., Knoops S.S. (1995). Multidimensional scaling of sorting data applied to cheese perception. Food Qual. Prefer..

[3] Dairou V., Sieffermann J.M. (2002). A comparison of 14 jams characterized by conventional profile and a quick original method, the flash profile. J. Food Sci..

[4] Pagès J. (2003). Direct collection of sensory distances: Application to the evaluation of ten white wines of the Loire Valley. Sci. Aliments.

[5] Adams J., Williams A., Lancaster B., Foley M. Advantages and uses of check-all-that-apply response compared to traditional scaling of attributes for salty snacks. Proceedings of the 7th Pangborn Sensory Science Symposium.

[6] Ares G., Bruzzone F., Vidal L., Cadena R.S., Giménez A., Pineau B., Hunter D.C., Paisley A.G., Jaeger S.R. (2014). Evaluation of a rating-based variant of check-all-that-apply questions: Rate-all-that-apply (RATA). Food Qual. Prefer..

[7] Varela P., Ares G. (2012). Sensory profiling, the blurred line between sensory and consumer science. A review of novel methods for product characterization. Food Res. Int..

[8] Lee S.M., Kim S.-E., Guinard J.-X., Kim K.-O. (2016). Exploration of flavor familiarity effect in Korean and US consumers’ hot sauces perceptions. Food Sci. Biotechnol..

[9] Moskowitz H.R. (1996). Experts versus consumers: A comparison. J. Sens. Stud..

[10] Stone H., Sidel J.L. (2004). Sensory Evaluation Practices.

[11] Stone H., Bleibaum R., Thomas H.A. (2012). Sensory Evaluation Practices.

[12] Costa A.I.A., Jongen W.M.F. (2006). New insights into consumer-led food product development. Trends Food Sci. Technol..

[13] De Pelsmaeker S., Gellynck X., Delbaere C., Declercq N., Dewettinck K. (2015). Consumer-driven product development and improvement combined with sensory analysis: A case-study for European filled chocolates. Food Qual. Prefer..

[14] Grunert K.G., Valli C. (2001). Designer-made meat and dairy products: Consumer-led product development. Livest. Prod. Sci..

[15] Jaeger S.R., Rossiter K.L., Wismer W.V., Harker F.R. (2003). Consumer-driven product development in the kiwifruit industry. Food Qual. Prefer..

[16] Linnemann A.R., Benner M., Verkerk R., van Boekel M.A.J.S. (2006). Consumer-driven food product development. Trends Food Sci. Technol..

[17] Prescott J., Monteleone E. (2015). Consumer perceptions of food and beverage flavour. Flavour Development, Analysis and Perception in Food and Beverages.

[18] van Kleef E., van Trijp H.C.M., Luning P. (2005). Consumer research in the early stages of new product development: A critical review of methods and techniques. Food Qual. Prefer..

[19] Næs T., Varela P., Berget I. (2018). Individual Differences in Sensory and Consumer Science.

[20] Tormod N., Brockhoff P.B., Tomic O. (2011). Statistics for Sensory and Consumer Science.

[21] Zaichkowsky J.L. (1986). Conceptualizing involvement. J. Advert..

[22] Kotler P., Keller K.L. (2009). Dirección de Marketing.

[23] Pliner P., Hobden K.L. (1992). Development of a scale to measure neophobia in humans the trait of food. Appetite.

[24] Eertmans A., Victoir A., Vansant G., Van den Bergh O. (2005). Food-related personality traits, food choice motives and food intake: Mediator and moderator relationships. Food Qual. Prefer..

[25] Jaeger S.R., Rasmussen M.A., Prescott J. (2017). Relationships between food neophobia and food intake and preferences: Findings from a sample of New Zealand adults. Appetite.

[26] Kim S.-E., Lee S.M., Kim K.-O. (2016). Consumer acceptability of coffee as affected by situational conditions and involvement. Food Qual. Prefer..

[27] Monteleone E., Spinelli S., Dinnella C., Endrizzi I., Laureati M., Pagliarini E., Sinesio F., Gasperi F., Torri L., Aprea E. (2017). Exploring influences on food choice in a large population sample: The Italian Taste project. Food Qual. Prefer..

[28] Zickgraf H.F., Schepps K. (2016). Fruit and vegetable intake and dietary variety in adult picky eaters. Food Qual. Prefer..

[29] Frandsen L.W., Dijksterhuis G.B., Martens H., Martens M. (2007). Consumer evaluation of milk authenticity explained both by consumer background characteristics and by product sensory descriptors. J. Sens. Stud..

[30] Frandsen L.W., Dijksterhuis G.B., Brockhoff P.B., Nielsen J.H., Martens M. (2007). Feelings as a basis for discrimination: Comparison of a modified authenticity test with the same–different test for slightly different types of milk. Food Qual. Prefer..

[31] Bell R., Marshall D.W. (2003). The construct of food involvement in behavioral research: Scale development and validation. Appetite.

[32] Williams E.J. (1949). Experimental designs balanced for the estimation of residual effects of treatments. Aust. J. Sci. Res..

[33] Marshall D., Bell R. (2004). Relating the food involvement scale to demographic variables, food choice and other constructs. Food Qual. Prefer..

[34] Zaichkowsky J.L. (1985). Measuring the involvement construct. J. Consum. Res..

[35] Stocks M., Shepherd D., Lee H.S., van Hout D., Hautus M.J. (2017). Cognitive decision strategies adopted by consumers in reminder difference tests: Influence of the authenticity test. Food Res. Int..

[36] Peter J.P., Olson J.C. (2010). Consumer Behavior & Marketing Strategy.

[37] Schiffman L., Kanuk L.L. (2010). Consumer Behavior, Global Tenth Edition.

[38] Solomon M.R. (2013). Consumer Behavior.

[39] Smith R.E., Swinyard W.R. (1983). Attitude-behavior consistency: The impact of product trial versus advertising. J. Mark. Res..

[40] Ares G., Besio M., Giménez A., Deliza R. (2010). Relationship between involvement and functional milk desserts intention to purchase. Influence on attitude towards packaging characteristics. Appetite.

[41] Raudenbush B., Frank R.A. (1999). Assessing food neophobia: The role of stimulus familiarity. Appetite.

[42] Frank R.A., Raudenbush B., Hoffman R.R., Sherrick M.F., Warm J.S. (1998). Individual differences in approach to novelty: The case of human food neophobia. Viewing Psychology as a Whole: The Integrative Science of William N. Dember.

[43] Brockhoff P.M., Skovgaard I.M. (1994). Modelling individual differences between assessors in sensory evaluations. Food Qual. Prefer..

[44] O’Mahony M. (1986). Sensory Evaluation of Food: Statistical Methods and Procedures.

[45] Romano R., Brockhoff P.B., Hersleth M., Tomic O., Næs T. (2008). Correcting for different use of the scale and the need for further analysis of individual differences in sensory analysis. Food Qual. Prefer..

[46] Brockhoff P.B., Schlich P., Skovgaard I. (2015). Taking individual scaling differences into account by analyzing profile data with the Mixed Assessor Model. Food Qual. Prefer..

[47] Brockhoff P.M. (1998). Assessor modelling. Food Qual. Prefer..

[48] Kuznetsova A., Christensen R.H., Bavay C., Brockhoff P.B. (2015). Automated mixed ANOVA modeling of sensory and consumer data. Food Qual. Prefer..

[49] Næs T., Langsrud Ø. (1998). Fixed or random assessors in sensory profiling?. Food Qual. Prefer..

[50] Næs T. (1990). Handling individual differences between assessors in sensory profiling. Food Qual. Prefer..

[51] Lim J. (2011). Hedonic scaling: A review of methods and theory. Food Qual. Prefer..

[52] Meilgaard M.C., Carr B.T., Civille G.V. (1999). Sensory Evaluation Techniques.

[53] Kähkönen P., Tuorila H. (1999). Consumer responses to reduced and regular fat content in different products: Effects of gender, involvement and health concern. Food Qual. Prefer..

[54] Choe J.Y., Cho M.S. (2011). Food neophobia and willingness to try non-traditional foods for Koreans. Food Qual. Prefer..

[55] Jeon S.-Y., Kim K.-O.K. (2018). Effect of portion size on long-term acceptability as affected by consumers’ neophobia level: A case study on flavored green-tea drinks. Food Qual. Prefer..

[56] Meiselman H.L., King S.C., Gillette M. (2010). The demographics of neophobia in a large commercial US sample. Food Qual. Prefer..

[57] Tuorila H., Lähteenmäki L., Pohjalainen L., Lotti L. (2001). Food neophobia among the Finns and related responses to familiar and unfamiliar foods. Food Qual. Prefer..

[58] Hursti U.-K.K., Sjödén P.-O. (1997). Food and general neophobia and their relationship with self-reported food choice: Familial resemblance in Swedish families with children of ages 7–17 Years. Appetite.

[59] Siegrist M., Hartmann C., Keller C. (2013). Antecedents of food neophobia and its association with eating behavior and food choices. Food Qual. Prefer..

[60] Koivisto U.-K., Sjödén P.-O. (1996). Food and general neophobia in Swedish families: Parent–child comparisons and relationships with serving specific foods. Appetite.

[61] Nordin S., Broman D.A., Garvill J., Nyroos M. (2004). Gender differences in factors affecting rejection of food in healthy young Swedish adults. Appetite.

[62] Olabi A., Najm N.E.O., Baghdadi O.K., Morton J.M. (2009). Food neophobia levels of Lebanese and American college students. Food Qual. Prefer..

[63] Pliner P., Melo N. (1997). Food neophobia in humans: Effects of manipulated arousal and individual differences in sensation seeking. Physiol. Behav..

[64] Mustonen S., Oerlemans P., Tuorila H. (2012). Familiarity with and affective responses to foods in 8–11-year-old children. The role of food neophobia and parental education. Appetite.

[65] Mustonen S., Tuorila H. (2010). Sensory education decreases food neophobia score and encourages trying unfamiliar foods in 8–12-year-old children. Food Qual. Prefer..

[66] Prescott J., Lee S.M., Kim K.O. (2011). Analytic approaches to evaluation modify hedonic responses. Food Qual. Prefer..

